# Thermoregulatory Instability in Childhood: Linking the Normal Brain to Hypothalamic Storm

**DOI:** 10.1155/2016/3903854

**Published:** 2016-10-26

**Authors:** William Alves Martins, Rafael do Amaral Cristovam, Helena Fussiger, Viviane Maria Vedana, Marta Hemb

**Affiliations:** ^1^Service of Neurology, Faculty of Medicine, PUCRS, Porto Alegre, RS, Brazil; ^2^Internal Medicine, Division of Neurology, Faculty of Medicine, Hospital São Lucas, Pontifícia Universidade Católica do Rio Grande do Sul (PUCRS), Porto Alegre, RS, Brazil

## Abstract

Central core temperature is tightly controlled by hypothalamic centers, a feature that makes sudden changes in body temperature very unusual. A dysfunction of these hypothalamic pathways leads to Shapiro's syndrome, comprising spontaneous hypothermia, hyperhidrosis, and corpus callosum dysgenesis. Although it may affect any age, usually it presents in childhood. Variants to this syndrome with completely normal brain anatomy have been consistently reported, expanding the clinical spectrum of the syndrome. Herein, we report the case of a 4-year-old girl with Shapiro's syndrome and unaffected corpus callosum.

## 1. Introduction

Central core temperature is tightly controlled by hypothalamic centers and sudden changes in body temperature are very uncommon [1]. Consequently, spontaneous periodic hypothermia (SPH) is rare, poorly understood, and frequently misdiagnosed [2]. When SPH combines with hyperhidrosis and corpus callosum agenesis (ACC), it receives the label of Shapiro's syndrome (SS) [3]. It has been proposed that a subtle malformation of midline prosencephalon may cause this thermoregulatory dysregulation [4]. Nonetheless, true pathophysiology remains a mystery to this date.

Atypical features are consistently reported, not making it any easier to recognize and treat this disease [2]. Usually, patients with Shapiro's syndrome may pass undiagnosed for years, as symptoms may wax and wane, especially patients presenting with SPH but without other signs of hypothalamic dysfunction and absent structural brain abnormalities. Nonetheless, ACC may not be a* sine qua non* condition for diagnosis, as originally defined, and SPH should thus be the main core feature, even without other abnormalities [3]. Herein, we describe a 4-year-old girl who developed SPH, hyperhidrosis, and other signs of autonomic dysfunction, but without any structural brain abnormalities, and review the current understanding of this unique syndrome.

## 2. Illustrative Case

A 4-year-old girl presented to our emergency room for recurrent attacks of unresponsiveness, pallor, and sweating. These fits were preceded by abdominal pain and vomiting. The patient remained hypotonic and drowsy for hours but was readily responsive under stimuli. There was no incontinence, but her mother noticed that, during these attacks, the patient would sweat enough to wet her entire bed. At 2 years of age, she had had four episodes, which had remitted spontaneously. She was born at 38 weeks in an uneventful pregnancy. Past medical history was unremarkable. Furthermore, family history was positive only for smoking parents and an aunt with benign childhood epilepsy.

**Figure 1:**
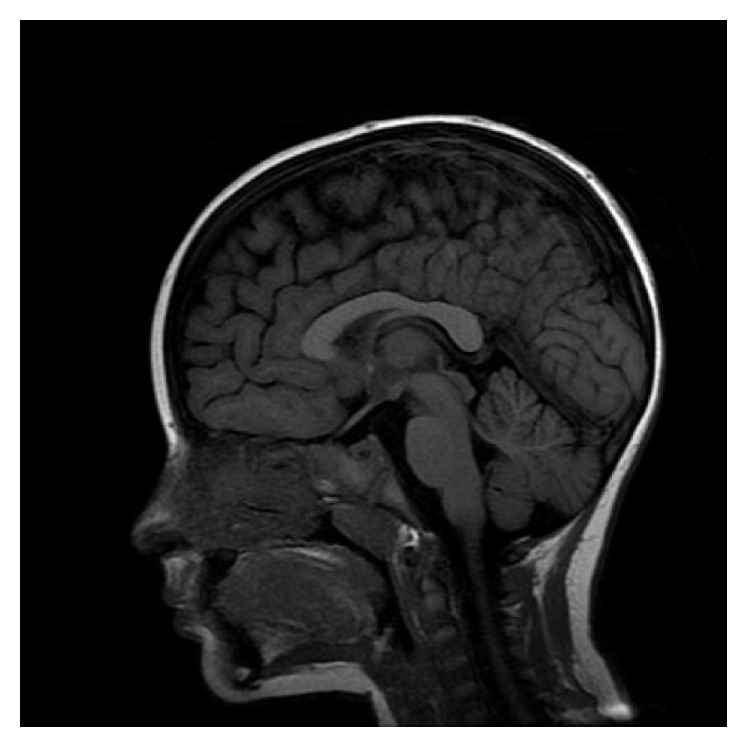
Brain MRI. Sagittal T1-weighted image demonstrating intact corpus callosum.

Three days before admission, she had a self-limited febrile illness. Physical examination was completely normal on arrival. Complete metabolic panel was normal, including renal and liver functions and electrolytes and endocrinological assessment. Cardiovascular studies were unremarkable. Electroencephalogram (EEG) did not reveal ictal or interictal epileptiform discharges. Screening for inherited errors of metabolism also was negative. Brain MRI was completely normal (Figure 1).

During admission, these paroxysmal events occurred 3 to 4 times a day, lasting from 30 minutes to several hours. Vital signs revealed rectal temperature of 34.3°C (93.74°F) during these events, associated with hypertension and mild bradycardia. Further investigation with video-EEG demonstrated slowing of background once these attacks began but did not record any ictal epileptiform activity. Ictal semiology was stereotyped, displaying disturbed awareness, pallor, sweating, bradycardia, and decreased body temperature, occasionally lasting up to 6 hours. Afterwards, she would promptly wake up and obey commands, as if nothing had happened.

Once epilepsy and metabolic and structural pathologies had been discarded, we established the diagnosis of SS and started her on clonidine. She had partial control of hypothermic spells and was discharged in good general condition, receiving lithium as an addition to clonidine. At the last follow-up, she had not experienced recurrence of the hypothermic spells and was being weaned off clonidine.

## 3. Discussion

Hypothermia (body temperature < 35°C) is a rather common physical finding, present in several conditions, such as infections, hypothyroidism, brain trauma, and tumors [1]. Conversely, spontaneous hypothermia is extremely rare and was recognized for the first time in 1969 by Shapiro et al., who reported two cases of SPH and hyperhidrosis accompanied by ACC, making these features the hallmark of this diagnosis [3]. Although 53 cases have been reported in the literature [2], a clear pathophysiological basis has not clearly been established.

Even though the triad of CCA, hypothermia, and hyperhidrosis is considered as the hallmark of Shapiro's syndrome, periodic hypothermia is the main link between this syndrome and its variants [2]. Congenital abnormalities of the corpus callosum are not specific for this syndrome and may even be associated with periodic* hyperthermia* [5]. Studying all cases in the literature, Tambasco et al. found that only half showed CC abnormalities [2]. In addition, Belcastro et al. described a familial case of SS in two siblings, where one had ACC whereas the other had a completely normal MRI, thus suggesting that callosal dysgenesis may be a marker of other molecular or microstructural abnormalities on hypothalamic networks [6]. Also of interest, acquired corpus callosum lesions have not been associated with any thermoregulatory dysfunction and do not seem to play any role in thermoregulation.

Shapiro's syndrome occurs across a wide range of ages, from 2 months to 90 years, a third of which presenting in childhood. As expected, SPH is the only feature present in all patients (100%), followed by hyperhidrosis (42.3%), pallor (23.1%), altered awareness (23.1%), and shivering (19.2%). Other signs of autonomic compromise may also be present, such as bradycardia (9.2%), arterial hypertension (7.7%), and cutaneous vasodilatation (7.7%) [2]. Periodicity of the spells is extremely variable, occurring any time from hours to weeks and lasting just a few minutes to several hours. Considering these clinical signs and symptoms alongside the absence of ACC in nearly half of the patients, a simplified approach to diagnose this syndrome would be to simply document SPH and autonomic dysfunction, keeping in mind that exclusion of other possible diagnoses is of paramount importance.

Hypothalamus is the central body “thermostat” and exerts control over shivering, peripheral vasomotricity, and sweating, keeping central core temperature under tight control [1]. Loss of heat by excessive sweat is one of the main mechanisms of hypothermia in SS. It is noteworthy that infections, nervous system malformations, trauma, tumors, and others may change the temperature setting up or down [1, 7]. In addition, hypothalamic lesions can cause sudden changes in body temperature [1]. Current theories state that small basal prosencephalic dysgenesis in SS may lead to spells of hypothermia [4]. Pathologic studies have revealed neuronal loss and fibrillary gliosis in the hypothalamus, infundibular nuclei, and severe spongiosis of the white matter [8]. Such hypothalamic alterations could therefore disrupt corticohypothalamic networks, leading to deregulation of the central thermostat and predisposition to paroxysmal autonomic instability. Spells would lead to massive release of plasmatic norepinephrine, explaining the partial response to clonidine and the frequent association of autonomic symptoms, such as pallor, hypertension, and bradycardia. Reflex bradycardia may explain the latter, as norepinephrine binds to alpha-2 receptors and the sudden hypertension that follows may stimulate baroreceptors in the carotid sinus [9]. Others have found high levels of melatonin, suggesting a possible role of this hormone [10]. Some considered this disease to be a form of diencephalic epilepsy, and although hypothalamic hamartomas and other dysplastic lesions are a known cause of subcortical epilepsy [11], there is no consistent evidence that SS shares this pathophysiology. This paroxysmal pattern of hypothalamic dysfunction is also a known feature of narcolepsy and Klein-Levin syndrome. Functional neuroimaging in SS has provided inconsistent results, even though PET scan showed hypermetabolism in the tectal plate regions, posterior pons, medulla, and cerebellar vermis, areas that could participate in body temperature regulation [12].

A thorough clinic-laboratory investigation must exclude other possible diagnoses and involve metabolic exams, cardiovascular analysis, EEG, and neuroimaging. Differential diagnosis of paroxysmal hypothermia includes congenital malformations, stroke, hypothalamic lesions, traumatic brain injury, hydrocephalus, and mitochondriopathies [1, 2, 12]. Because of the episodic nature and eventual clouding of awareness of SS, exclusion of epileptic disorders with autonomic features is indispensable. Of these, Panayiotopoulos syndrome deserves special consideration, as ictal manifestations involve vomiting, unresponsiveness, and other striking autonomic manifestations [13]. Mostly occurring in young children, hypothermia is not a consistent finding, making differentiation easier.

Treatment relies solely on evidence of case reports. A range of different medications have been tried, including sympatholytics (clonidine), antiserotonergic agents (cyproheptadine), dopaminergic drugs (levodopa and bromocriptine), anticholinergics (glycopyrrolate), anticonvulsants (carbamazepine, phenytoin, valproate, and phenobarbital), and even botulinum toxin [2, 14]. This reflects our lack of understanding of this syndrome's pathophysiological process. So far, clonidine and cyproheptadine are regarded as the most effective. Clinical response to the latter suggests a central serotoninergic dysfunction in addition to disruption of hypothalamic networks [15]. Our decision to use lithium was based on its use in other neurological syndromes of hypothalamic dysfunction, particularly Klein-Levin syndrome and cluster headache [16], and this may be a novel therapeutic approach, since the combination of lithium and clonidine rendered our patient “hypothermia-free.” Evolution is usually benign, but premature death and even renal failure had been reported. Nevertheless, some patients remain with residual hypothermic spells or even worsen over time, irrespective of therapy.

In conclusion, clinical awareness about this remarkable syndrome and its variants is required for diagnosis, proper treatment, and prognostication. Through each new patient, a small portion of this mysterious syndrome is unveiled and may help to further comprehend both the dysregulation and the function of the thermoregulatory system.
